# The Attitude of Some Short-Horn Breeders toward the Tuberculin Test, and the Animus Thereof

**Published:** 1901-02

**Authors:** 


### EDITORIALS.

### THE ATTITUDE OF SOME SHORT-HORN BREEDERS TOWARD THE TUBERCULIN TEST, AND THE ANIMUS THEREOF.

Some Short-horn breeders have held a meeting in Kansas City. The meeting was a notable one, because during it occurred the outbreaking of the terrific storm, the gathering clouds of which have been seen from afar and have been heralded loudly, and even hysterically, by a certain *Journal for the American Stock Farmer*, published in Chicago. The storm took the shape of the resolution that is printed in full below :

Whereas, The tuberculin test of breeding animals now required by the quarantine regulations of the United States and Canada is proving hurtful to the cattle-breeding industry of America ; and

Whereas, Experience has shown that the test is not an infallible agent to determine the presence of tuberculosis ; that it is in no manner a preventive of the future development of tuberculosis in animals tested ; that it is frequently productive of serious constitutional derangement in otherwise healthy animals ; and

Whereas, While proving no substantial protection to our herds it operates as a serious barrier to the exchange of breeding animals between different sections, and in many ways is hurtful to the cattle-breeding industry ; therefore, be it

*Resolved*, That we respectfully petition the honorable Secretaries of Agriculture for the United States and Canada and the State Sanitary Boards of the United States to so modify the quarantine regulations of their departments that cattle breeders may be relieved of this menace to their herds and unnecessary restriction upon their business.

*Resolved*, That the Secretary of this Association be instructed to forward a copy of these resolutions to the Ministers of Agriculture for the United States and Canada and the Livestock Sanitary Board of every State in the Union.

[Full text of a resolution adopted by the Central Short-horn Breeders' Association at their annual meeting in Kansas City, Mo., January 29 and 30, 1901.]

This is gravely announced by the aforementioned journal as the cyclone that is to wipe out the tuberculin test.

What a disappointment ! We were preparing to hear some real objections to the use of tuberculin ; but, instead of heavy guns loaded with real charges, here are arrayed the shades of the objections used ten years ago, and dead nearly as long, revamped, newly decorated, and as impressive and terrifying as painted paper Chinese dragons.

The mountain has groaned and quaked and has brought forth —a mouse.

Does it mean that the Short-horn breeders are afraid to have their cattle tested? If so, does not this mean that their herds are tubercular? Then, does it mean that they wish to have the unrestrained privilege of spreading disease and infecting other herds?

These questions have been asked by a sincere admirer of Short-horn cattle. They are reproduced here with a full knowledge of the enormous benefit Short-horns have been to this country and with the most profound regret that a vast number of breeders of these grand cattle have been placed in a false light by a few of their representatives at the meeting in Kansas City.

Short-horn breeders should approach the tuberculosis problem with a full sense of its importance. Their experience with this disease should cause them to have a feeling of deep responsibility in discussing it. They should adopt a judicial attitude and listen carefully to both sides of the question. They should not forget that there is the side of the man who wishes to sell tubercular cattle and the side of the man who wishes to buy sound cattle. There is the side of the Central Short-horn Breeders in convention assembled and the side of the other 74,000,000 Americans who have determined that the ravages of tuberculosis shall be checked.

If the public looks askance at the Short-horn Breeders' resolution they will have many facts of history to support them, of which the following are a few:

Tuberculosis was taken to the Argentine Republic by Short-horn cattle and is now spreading at an alarming rate.

Tuberculosis is reported to have been taken to Japan by Short-horn cattle.

Tuberculosis is believed by those who have investigated the subject most carefully to have been started among the cattle of Denmark by Short-horn cattle.

Extensively tubercular herds of Short-horns have been found and reported in France, Germany, and Austria.

Both Bang and Nocard have reported extensively infected Short-horn herds in their countries.

The percentage of tuberculosis among the cattle of England is higher among Short-horns than among cattle of any other breed.

The first extensively tubercular herd in New York State—a herd that was finally wiped out by this disease—was a famous herd of Short-horns. These cattle scattered disease widely.

The worst infected herd that has been examined by the Agricultural Department of Pennsylvania was a herd of pure-bred and grade Short-horns. Of 164 cattle in this herd 156 were condemned.

A large herd of Short-horn cattle in Ohio has recently been found to be tubercular and has been killed by the owner, voluntarily and without indemnity.

Numerous tubercular herds of Short-horns, pure-bred and grade, have been reported from Iowa, Illinois, Michigan, and Indiana.

Tubercular Short-horn steers are found by meat inspectors in abattoirs all over the United States.

Tubercular Short-horns from England have been landed in Canada and have died of this disease within a short time after arrival.

We regret having to give publicity to these facts, and do so only to aid our readers to truly interpret and understand the animus of the resolution.

Now, as to the allegation that the tuberculin test is injurious. *This is a subterfuge.* Ten years ago, before the test was well known, it was natural that people should fear that it might be harmful; but now that thousands and hundreds of thousands of cattle have been tested in this country and in every civilized country in the world, and there is no evidence of any harm whatever, the allegation is infantile, flat, and absurd.

Of course, it has happened that cattle have aborted, or have lost condition, or have broken their legs, or have died following the test. But did cattle never abort, or lose condition, or break their legs, or die before the test was invented, and are untested herds immune to such accidents? To say that such and such things have followed the test is not to say that they were due to the test. It has never been shown, and there is no reason to believe, that accidents and illness are any more common, or indeed as common, in tested as in untested herds.

Agricultural Experiment Stations all over the country have tested their herds. Have they observed any injury? No. Government commissions all over the world have investigated this subject. Have they found the test harmful? No. Many of the best herds in the United States have been tested. Have they been injured? Evidently not, for their owners continue to test cattle purchased. The test has been widely employed in many localities in the United States. Has injury been noticed? On the contrary, the herds in these localities are improved in condition and the test is growing in use most rapidly in such places.

It is necessary, of course, that the tuberculin shall be reliable and clean and that it shall be used by men who understand it. We are here discussing the tuberculin test and not the misuse of tuberculin by ignoramuses. Misuse of anything is harmful. A cow may be killed by pouring flour and water into her mouth from a bottle if her head is held too high and the fluid goes into the windpipe, yet this need not be and is not considered a harmful operation; nor is the tuberculin test, properly used, more harmful than a dose of flour and water.

The Short-horn breeders object that the test is not infallible. True, the test is not infallible—nothing is—but it is reliable. It furnishes immeasurably the most reliable means of recognizing tuberculosis in the living animal, and no other disease can be diagnosed with the accuracy that tuberculosis can be with the aid of tuberculin.

By making this ill-considered attack upon the tuberculin test—an attack founded manifestly upon false premises, and, therefore, ascribable to motives they dared not express—the Short-horn breeders have diametrically opposed their own best interests. They have attempted to discourage the use of tuberculin, and this means that they encourage the harboring of tubercular cattle in their herds; for even the *Breeders' Gazette* admits that tuberculin has a place in private veterinary practice.

Some day the Short-horn breeders will wake up, take stock of their diminished assets, and look back with wide-eyed wonder upon the time that they were led astray by Canadians at Kansas City. They will then wonder how it was possible for them to have been enticed into the false attitude of issuing a special plea for a chance to propagate and distribute tuberculosis.

